# Regional Anesthesia: A Narrative Review of Impact on Oxidative Stress Biomarkers

**DOI:** 10.3390/jcm14217503

**Published:** 2025-10-23

**Authors:** Karolina Jaruga, Anna Puścion-Jakubik, Piotr Jakubów

**Affiliations:** 1 Department of Anesthesiology and Intensive Care for Children and Adolescents with Postoperative Care and Pain Management Unit, Medical University of Białystok, 15-274 Białystok, Poland; piotr.jakubow@umb.edu.pl; 2 Department of Bromatology, Faculty of Pharmacy with the Division of Laboratory Medicine, Medical University of Białystok, 15-222 Białystok, Poland; anna.puscion-jakubik@umb.edu.pl

**Keywords:** oxidative stress, anesthesiology, regional anesthesia, oxidative stress biomarkers

## Abstract

**Background/Objectives**: Oxidative stress results from an imbalance between reactive oxygen species (ROS) production and antioxidant defenses and has been implicated in the pathogenesis of numerous diseases, including cardiovascular and neurodegenerative disorders. In the perioperative setting, both surgical trauma and anesthetic agents may contribute to oxidative stress. While this phenomenon has been extensively studied under general anesthesia, the role of regional anesthesia remains less defined. This review aims to compare the impact of general and regional anesthesia techniques on oxidative stress and its biomarkers across various surgical disciplines. **Methods**: A literature search was conducted using PubMed and EBSCO databases, focusing on studies published between 2010 and 2024. Search terms included “anesthesiology”, “oxidative stress”, “regional anesthesia”, “general anesthesia”, and related biomarkers (e.g., MDA, TAS, TOS, thiol/disulfide). Inclusion criteria required that studies compare general and regional anesthesia techniques and assess at least one biomarker of oxidative stress. Seventeen studies were included in the final analysis. **Results**: Findings were heterogeneous. Some studies reported that regional anesthesia, particularly spinal and peripheral nerve blocks, was associated with lower levels of malondialdehyde (MDA), reduced total oxidant status (TOS), and improved total antioxidant status (TAS), suggesting reduced oxidative stress. In contrast, other studies showed higher levels of oxidative stress markers under regional anesthesia or no significant differences between techniques. Outcomes varied by surgical type, biomarker used, patient population, and methodological quality. **Conclusions**: Current evidence is insufficient to establish definitive recommendations regarding the choice of anesthetic technique based on oxidative stress outcomes. However, regional anesthesia appears to be associated with a more favorable oxidative profile in some settings, such as cesarean section and selected orthopedic procedures. Further standardized, large-scale studies are needed to clarify these findings and guide anesthetic decision-making in the context of oxidative balance.

## 1. Introduction

Oxidative stress is a pathological condition defined by the disruption of redox signaling and control due to an imbalance between reactive oxygen species (ROS) production and the cell’s antioxidative defense [1]. Reactive oxygen species (ROS) are highly reactive molecules generated during the metabolism of oxygen or nitrogen. They include both free radicals, such as superoxide (O_2_•^−^), hydroxyl radical (•OH), and nitric oxide (NO•), as well as non-radical species like hydrogen peroxide (H_2_O_2_) and peroxynitrite (ONOO^−^) [2].

These molecules induce oxidative damage to proteins, lipids, and DNA, leading to the disruption of cellular structures and impairment of their functions, thereby contributing to the onset and persistence of inflammatory processes [3]. Research indicates that oxidative stress plays a key role in the development of many pathological conditions such as diabetes, cancer, cardiovascular disorders as well as neurodegenerative diseases [4]. Knowledge about the harmful effects of oxidative stress on human health is required, as anesthesiologists must consider patients’ oxidative status when selecting the safest possible anesthesia method [5]. Numerous factors influence oxidative stress in the context of anesthesia. It is not determined solely by the pharmacological agents or anesthetic techniques employed, but is also significantly affected by the type and extent of the surgical procedure, as well as by the patient’s preoperative physiological and metabolic status. Sources of oxidative stress connected to anesthesiology include surgical tissue trauma, the process of ischemia/reperfusion injury due to interruption of blood flow with a tourniquet (e.g., in orthopedic anesthesia) or inadequate tissue perfusion. Another factor involves anesthetic agents, as certain intravenous or inhalational anesthetics can promote free radical formation. On the other hand, there are studies demonstrating antioxidant properties of some intravenous anesthetics such as propofol [6,7].

Although oxidative stress has been predominantly investigated in the context of general anesthesia and intensive care, growing attention is being paid to regional anesthesia and the potential relationship between local anesthetics and the induction of oxidative stress or its antioxidant properties [8,9,10]. The aim of this review is to analyze studies comparing regional anesthesia and general anesthesia in the context of the correlation between them and oxidative stress and its biomarkers.

## 2. Materials and Methods

To collect materials for the review article, the PubMed database and the EBSCO databases provided by the Medical University of Białystok Library were employed. The analysis focused on articles published from 1 January 2010 to 31 August 2025. Initially, studies published within the last 10 years were planned for inclusion; however, the time frame was later extended to 15 years to increase the number of eligible articles and provide a more comprehensive overview of the available evidence. The search phrases ‘anesthesiology’, ‘oxidative stress, and ‘regional anesthesia’, ‘general anesthesia’, ‘MDA’, ‘Malondialdehyde’, ‘TAS’, ‘TOS’, ‘thiol’ were used in various combinations using Boolean operators (AND, OR). The review included articles reporting randomized controlled trials that fulfilled the following criteria:

1. In the study, patients were undergoing surgical procedures (general surgery, orthopedic, obstetric/gynecologic), both adults and children. In addition to clinical studies in adults and children, relevant animal studies were included to broaden the evidence base and provide supplementary mechanistic data for analysis.
2. The study was based on a comparison of general anesthesia (including inhalation anesthesia, TIVA, or mixed techniques) with regional anesthesia (such as epidural anesthesia, spinal anesthesia, or other regional anesthesia techniques).
3. At least one biomarker of oxidative stress was evaluated in the study. One of the studies included in this review investigated gene expression profiles related to oxidative stress, focusing on the expression of antioxidant enzymes such as superoxide dismutase (SOD) and glutathione peroxidase (GPX). Twenty-one research articles comparing general anesthesia and regional anesthesia in the context of their impact on oxidative stress parameters were found. To facilitate comparison and ensure systematic presentation, the results were organized into sections corresponding to surgical disciplines (orthopedics, general surgery, obstetrics/gynecology) and animal models, allowing evaluation of similar patient groups within each category.

## 3. Biomarkers of Oxidative Stress

The assessment of oxidative stress can be approached through the measurement of various biomarkers. These include oxidative damage biomarkers that are generated during oxidative processes, antioxidant enzyme activities reflecting the body’s defensive capacity, and global indices such as the Total Antioxidant Status (TAS), Total Oxidant Status (TOS), and the Oxidative Stress Index (OSI), which together provide an integrated evaluation of the overall redox balance. Oxidative stress biomarkers can be measured in various biological samples, depending on the study design and the target organ system. They are most commonly determined in blood (serum or plasma), but can also be assessed in urine, saliva, exhaled breath condensate, cerebrospinal fluid, or tissue homogenates [11,12]. In studies on oxidative stress in anesthesiology, the most common biomarkers of oxidative stress include those shown in Table 1.

### 3.1. Products of Oxidative Damage

(a) Lipid Peroxidation Products

Malondialdehyde (MDA) is a reactive aldehyde formed as a byproduct of lipid peroxidation of polyunsaturated fatty acids. It is one of the most widely used biomarkers of oxidative stress, reflecting damage to cell membrane lipids. F_2_-Isoprostanes are stable compounds formed non-enzymatically through the free radical-induced peroxidation of arachidonic acid in cell membrane phospholipids. F_2_-Isofurans are oxidative lipid metabolites produced, like F_2_-Isoprostanes, from arachidonic acid, but their formation is favored under conditions of high oxygen tension. Plasma levels of isoprostanes and isofurans (F2-IsoPs and IsoFs) can be determined using gas chromatography–mass spectrometry (GC-MS) [13].

(b) Protein Oxidation Products

Protein Carbonyls are oxidative modifications of proteins that occur when reactive oxygen species (ROS) attack amino acid side chains—particularly those of lysine, arginine, proline, and threonine—leading to the introduction of carbonyl (C=O) groups into the protein structure. Protein carbonyls can be measured spectrophotometrically or by ELISA after derivatization with 2,4-dinitrophenylhydrazine (DNPH), which forms hydrazone derivatives detectable at 370–380 nm [14,15].

Total Thiols (TTs) represent the overall concentration of sulfhydryl (–SH) groups in biological samples, which play a crucial role in maintaining the redox balance and antioxidant defense of the organism.

Thiol groups are primarily found in proteins (especially cysteine residues) and in low-molecular-weight compounds such as glutathione (GSH), cysteine, and homocysteine. They act as electron donors, neutralizing reactive oxygen species (ROS) and repairing oxidized biomolecules.

Measurement of total thiols provides an integrated marker of antioxidant capacity. The most commonly used method involves the reaction with Ellman’s reagent (5,5′-dithiobis-(2-nitrobenzoic acid), DTNB), producing a yellow-colored compound (TNB) measurable spectrophotometrically [16].

Native Thiols (NTs) refer to the fraction of free, unoxidized sulfhydryl (–SH) groups present in biological samples. They represent the reduced form of thiols that are directly available to counteract reactive oxygen species (ROS) and maintain cellular redox homeostasis. In physiological conditions, thiol groups can undergo reversible oxidation to form disulfide bonds (–S–S–) during oxidative stress. Therefore, the measurement of native thiols, together with total thiols (TT) and disulfides (SS), provides valuable information about the thiol–disulfide homeostasis, a dynamic indicator of oxidative balance.

Native thiol levels are most commonly determined using the automated spectrophotometric method.

Disulfide Levels (DS) refer to the concentration of oxidized thiol groups that form disulfide bonds (–S–S–) between two cysteine residues in proteins or low-molecular-weight thiols. Disulfides are generated when native thiols (–SH) are oxidized during oxidative stress, and they can be reduced back to thiols, reflecting the dynamic thiol–disulfide homeostasis.

Thiol–Disulfide Ratio (NT/DS): the equilibrium between antioxidant defenses and oxidative processes. A higher NT/DS ratio reflects a predominance of reduced thiols and a stronger antioxidant capacity, whereas a lower NT/DS ratio indicates oxidative shift, meaning increased oxidation of thiols into disulfides.

This ratio is typically derived from measurements of native thiols (NT), total thiols (TT), and calculated disulfides (DS = (TT − NT)/2).

### 3.2. Indicators of Antioxidant Status

Catalase (CAT) is a key antioxidant enzyme that catalyzes the decomposition of hydrogen peroxide (H_2_O_2_) into water (H_2_O) and oxygen (O_2_), thereby protecting cells from oxidative damage caused by excessive reactive oxygen species (ROS).

CAT is predominantly located in peroxisomes, and to a lesser extent in cytoplasm and mitochondria. It acts in coordination with other antioxidant enzymes such as superoxide dismutase (SOD) and glutathione peroxidase (GPX) to maintain redox balance within the cell. The activity of catalase can be measured spectrophotometrically, based on the rate of H_2_O_2_ decomposition (usually monitored at 240 nm), or by colorimetric and fluorometric assays.

Glutathione Peroxidase (GPX) is a major selenium-dependent antioxidant enzyme that protects cells from oxidative damage by catalyzing the reduction of hydrogen peroxide (H_2_O_2_) and organic hydroperoxides into water (H_2_O) and corresponding alcohols. During this process, reduced glutathione (GSH) is oxidized to glutathione disulfide (GSSG).

GPX plays a central role in maintaining cellular redox homeostasis and works synergistically with glutathione reductase (GR), which regenerates GSH from GSSG using NADPH.

The activity of GP is most commonly measured spectrophotometrically by monitoring the oxidation of NADPH to NADP^+^ at 340 nm in coupled enzyme assays.

### 3.3. Global Oxidative Stress Indicators

Total Antioxidant Capacity (TAC) represents the overall ability of all antioxidants in a biological sample to scavenge or neutralize reactive oxygen species (ROS). It reflects the cumulative action of both enzymatic antioxidants (such as superoxide dismutase, catalase, and glutathione peroxidase) and non-enzymatic antioxidants (including vitamins C and E, uric acid, bilirubin, albumin, and thiol groups). TAC provides a general estimate of the total reducing power of plasma or tissues. It can be measured by several assays—most commonly FRAP (Ferric Reducing Ability of Plasma), ABTS, ORAC, or DPPH—and results are typically expressed as Trolox equivalents [17].

Total Antioxidant Status (TAS) quantifies the actual antioxidant potential of serum or plasma at a given time point and is often used as a clinical biomarker of systemic redox balance. The most frequently used method is the automated ABTS-based colorimetric assay developed by Erel (2004) [18]. TAS reflects the integrated activity of both plasma and cellular antioxidants and is expressed in mmol Trolox equivalents per liter [18].

Total Oxidant Status (TOS) measures the overall oxidant load in a biological sample, encompassing all oxidizing molecules such as reactive oxygen and nitrogen species (ROS, RNS), lipid peroxides, and hydroperoxides. It represents the oxidative counterpart to TAS and provides a quantitative estimate of pro-oxidant activity. TOS is usually determined using the Erel method (2005) [19], which relies on the oxidation of ferrous ions to ferric ions by oxidants present in the sample, with results expressed in μmol H_2_O_2_ equivalents per liter [19].

Oxidative Stress Index (OSI) integrates both antioxidant and oxidant measurements to express the overall redox balance. It is calculated as the ratio of TOS to TAS, multiplied by 100. A higher OSI value indicates a predominance of oxidative processes and thus an increased level of oxidative stress, whereas a lower OSI reflects a more favorable antioxidant status.

## 4. Oxidative Stress Across Surgical Disciplines

### 4.1. General Surgery

Regional anesthesia (including spinal anesthesia and peripheral nerve blocks), due to its significant advantages such as reduced opioid consumption, improved postoperative pain control, and a lower risk of postoperative nausea and vomiting (PONV), has become an interesting alternative to general anesthesia. Additionally, it is associated with fewer respiratory complications and faster recovery, making it worth considering as an option in certain surgical procedures [20,21].

Referring to oxidative stress, the comparison of the influence of regional and general anesthesia techniques on oxidative status remains inconclusive. Several articles related to the research conducted in Finland that studied the relationship between rectus-sheath block (single dose, repeated dose and continuous-infusion) and oxidative stress before and after midline laparotomy in patients with Cancer and Benign Disease showed significant postoperative increase in plasma SOD levels (*p* = 0.007) with normalization at 24 h, and a trend toward inverse correlation with pain scores (r = −0.30, *p* = 0.09), suggesting possible antioxidative and analgesic interactions. Study found no significant effect of RSB on plasma 8-OHdG levels, though 8-OHdG correlated inversely with hs-CRP (r = −0.40, *p* = 0.02), indicating a link between oxidative stress and inflammation. GPX levels decreased after surgery in all groups, with the lowest levels in the single-dose RSB group (*p* = 0.032); cancer patients showed significantly lower baseline GPX than those with benign disease (*p* = 0.006). Overall, RSB did not consistently reduce oxidative stress markers but may modulate redox response depending on dose and disease status [22,23,24].

Considering a correlation between anesthesia methods and dynamic thiol/disulfide homeostasis, a recent study on patients with Chronic Obstructive Pulmonary undergoing inguinal hernia surgery under general and spinal anesthesia showed that general and spinal anesthesia induce similar oxidative stress responses in COPD patients. Both anesthesia types resulted in a postoperative decrease in total and native thiol levels, but this reduction was statistically significant only in the SA group (*p* < 0.01 and *p* = 0.012, respectively). However, no significant differences were observed between GA and SA in postoperative thiol/disulfide ratios [25].

A beneficial effect of combined general and epidural anesthesia on oxidative stress was demonstrated in a randomized controlled study by Shin et al. (2013) [26]. The study included 38 patients undergoing robot-assisted laparoscopic prostatectomy and was conducted in South Korea. The authors compared general anesthesia (G, *n* = 19) with combined general and epidural anesthesia (GE, *n* = 19), focusing on oxidative stress and autonomic nervous system activity. Oxidative stress was evaluated by measuring serum malondialdehyde (MDA) and plasma nitrite (NO_2_^−^) levels before and after pneumoperitoneum.MDA levels significantly increased in the G group after pneumoperitoneum, indicating elevated lipid peroxidation, whereas they remained stable in the GE group. Similarly, plasma nitrite levels decreased in the G group but were preserved in the GE group, suggesting that epidural co-administration helps maintain nitric oxide availability and mitigates oxidative damage during laparoscopic surgery [26].

### 4.2. Orthopedics Surgery

Orthopedic surgeries are the type of procedures in which regional anesthesia is most frequently utilized. The variety of techniques and blocks allows the safe performance of most upper and lower limb surgeries. The subject of advantages, safety and limitations of this type of anesthesia has been widely investigated but there is limited research about its impact on oxidative stress [27].

Koşucu et al. (2014) [28] conducted a randomized clinical study comparing the effects of spinal, inhalational, and total intravenous anesthesia (TIVA) on biochemical markers of ischemia–reperfusion injury (IRI) in patients undergoing arthroscopic knee surgery with tourniquet application. Sixty ASA I–II patients were randomized to receive spinal anesthesia with levobupivacaine, inhalation anesthesia with sevoflurane, or TIVA with propofol. Plasma concentrations of malondialdehyde (MDA) and ischemia-modified albumin (IMA)—markers of oxidative stress and tissue ischemia—were measured before induction, during ischemia, and up to 6 h after tourniquet release. The study demonstrated a significant rise in both MDA and IMA levels in the spinal anesthesia group, indicating greater oxidative stress. In contrast, both sevoflurane and propofol-based TIVA attenuated the increase in these biomarkers, with propofol showing the strongest protective effect. MDA levels in the TIVA and inhalation groups were significantly lower than in the spinal group at nearly all post-ischemic time points, and IMA levels were significantly reduced with TIVA compared to spinal anesthesia. Minor transient motor deficits were observed in a few patients, but without lasting neurological impairment. The authors concluded that TIVA with propofol confers measurable antioxidant protection during tourniquet-induced ischemia–reperfusion in arthroscopic knee surgery, possibly through free radical scavenging similar to vitamin E activity. Sevoflurane also appeared to provide partial protection, whereas spinal anesthesia alone was associated with the highest oxidative stress response [28].

A similar conclusion was made in research investigating different biomarkers of oxidative stress in knee surgery, where the decrease in thiol levels and the increase in disulfides were considered indicators of oxidative stress. Şimşek et al. (2023) [29] conducted a randomized comparative study to evaluate how spinal versus general anesthesia influences oxidative stress—assessed by thiol–disulfide homeostasis—in patients undergoing total knee replacement with tourniquet application. Fifty-six ASA I–II patients aged 60–74 years were randomized to receive either general anesthesia (propofol induction with sevoflurane/remifentanil maintenance) or spinal anesthesia with bupivacaine. Blood samples were collected before anesthesia (T1), at 5 (T2) and 40 min (T3) after tourniquet release, and at 24 h postoperatively (T4). Levels of native thiol, total thiol, and disulfide and their ratios (disulfide/native thiol, disulfide/total thiol, and native thiol/total thiol) were analyzed using the Erel–Neselioğlu spectrophotometric assay. Compared with spinal anesthesia, the general anesthesia group showed significantly higher native and total thiol concentrations and lower disulfide levels and disulfide ratios at almost all time points (T1–T4), suggesting a more favorable redox balance. Within-group analysis revealed that thiol–disulfide indices changed significantly over time only in the spinal anesthesia group, indicating greater oxidative perturbation during ischemia–reperfusion. Postoperative pain scores (VAS) were higher initially in the general anesthesia group but equalized by 24 h, and there were no differences in tourniquet time or hemodynamic parameters between groups. The authors concluded that general anesthesia attenuates tourniquet- and surgery-induced oxidative stress more effectively than spinal anesthesia, likely due to the antioxidant effects of propofol, sevoflurane, and remifentanil, which may stabilize thiol–disulfide homeostasis. The study reinforces evidence that systemic anesthetic agents with intrinsic antioxidant properties confer measurable biochemical protection against ischemia–reperfusion injury during orthopedic surgery [29].

Other biomarkers of oxidative stress evaluated in knee surgery were F2-Isoprostanes (F2-IsoPs) and Isofuranes (IsoFs). These biomarkers were measured by gas chromatography–mass spectrometry at baseline and 30 min, 2 h, and 24 h after tourniquet release. The study showed that plasma F_2_-IsoP concentrations were significantly higher in the spinal anesthesia group compared with general anesthesia group (*p* = 0.045), indicating greater lipid peroxidation under lower tissue oxygenation. Conversely, IsoF levels were higher during general anesthesia (*p* = 0.032). Authors suggest that the magnitude of IsoFs in general anesthesia group can be explained by increased oxygen exposure during the procedure [30].

In contrast to these findings from a trial conducted in Iran in 2013, the effects of spinal versus general anesthesia on oxidative stress were evaluated in 40 diabetic patients undergoing lower limb amputation. These patients were initially exposed to oxidative stress due to diabetes. Oxidative stress was assessed by measuring serum levels of malondialdehyde (MDA) and total antioxidant capacity (TAC) before anesthesia and one hour after surgery. Both anesthesia techniques led to a reduction in MDA and an increase in TAC postoperatively. However, spinal anesthesia resulted in a significantly lower postoperative MDA level (19.24 ± 2.7 µM vs. 23.14 ± 2.6 μM, *p* = 0.03) and higher TAC compared to general anesthesia. These results suggest that spinal anesthesia may offer superior control of oxidative stress in diabetic patients undergoing amputation, likely due to reduced systemic redox imbalance [31].

The results of a randomized clinical study comparing the effects of general anesthesia (GA) and spinal anesthesia (SA) on oxidative stress and inflammation in 40 orthopedic patients undergoing open reduction and internal fixation (ORIF) also demonstrated that SA was associated with a more favorable oxidative stress profile compared with general anesthesia GA. The authors measured several oxidative stress biomarkers—malondialdehyde (MDA) as an index of lipid peroxidation, reduced glutathione (GSH) as a key intracellular antioxidant, and catalase (CAT) activity as an enzymatic defense marker—before and after surgery. The results showed a significant postoperative increase in serum MDA levels (*p* = 0.001) and a significant decrease in catalase activity (*p* = 0.017) in patients who received general anesthesia, whereas no such changes were observed in the spinal anesthesia group. Both GSH and nitrite concentrations remained stable in both groups. These findings indicate that general anesthesia induced a stronger oxidative response, characterized by enhanced lipid peroxidation and reduced antioxidant enzyme activity. Mechanistically, the authors attributed this increase in oxidative stress under GA to the release of cortisol and catecholamines during surgical stress, which promotes the generation of ROS and lipid radicals. The combination of thiopental, fentanyl, and halothane used in GA was also discussed as a possible contributor to ROS production via redox cycling mechanisms. Conversely, the spinal technique with bupivacaine appeared to minimize oxidative disturbance, likely by reducing neuroendocrine activation and systemic oxygen demand [32].

In randomized, double-blind clinical trial, Zhao and Qiu (2024) [33] investigated whether ultrasound-guided femoral nerve block (FNB) could reduce postoperative delirium (POD) and oxidative stress after total knee arthroplasty (TKA) under general anesthesia in elderly patients. A total of 297 patients aged 65–80 years were randomized to receive either standard general anesthesia (NC group) or general anesthesia combined with FNB (FNB group). To assess oxidative stress and inflammation, plasma levels of MDA and GPX were measured together with CRP before surgery, intraoperatively, and on postoperative days 1 and 3. The study demonstrated that patients receiving FNB had significantly lower postoperative MDA levels and higher GPX activity than those in the control group both on day 1 and day 3 after surgery (*p* < 0.001 for both comparisons). These results indicate that FNB effectively attenuated systemic oxidative stress induced by surgical trauma and ischemia–reperfusion during TKA. The reduction in oxidative stress was accompanied by a lower incidence of postoperative delirium (21% vs. 32%; *p* = 0.038) and a better early recovery quality (higher QoR-15C scores on day 5; *p* = 0.042). These results indicate that ultrasound-guided femoral nerve block significantly reduced postoperative oxidative stress [33].

Interesting findings about gene expression profiles in 99 patients who underwent hip arthroplasty with the application of various anesthesia techniques were demonstrated in Italian study from 2019. The comparison of lumbar plexus block with spinal anesthesia, general anesthesia and integrated anesthesia (the combination of these two techniques) showed that regional anesthesia had the least impact on analyzed gene expression. However the integrated anesthesia resulted in the most extensive changes in expression of genes responsible for DNA repair (ERCC3, RAD50, XRCC1), oxidative stress response (SOD, GPX) and cell damage response (EGR1, HSF1), which persisted for up to three days postoperatively [34].

When it comes to upper limb surgery, studies indicate a beneficial effect of brachial plexus block on oxidative balance. A trial conducted in 2021 compared the effects of general anesthesia (GA) and ultrasound-guided interscalene block (IB) on oxidative stress in patients undergoing shoulder arthroscopy. Forty-two ASA I–II adults were randomized to receive either GA (*n* = 22) with propofol, sevoflurane, and remifentanil, or IB (*n* = 20) with bupivacaine + lidocaine. Oxidative stress was assessed using thiol–disulfide homeostasis measured by the Erel and Neselioglu spectrophotometric method. Blood samples were taken intraoperatively and at 3, 6, and 18 h postoperatively.

Native thiol and total thiol concentrations were significantly higher in the IB group, while disulfide and disulfide/native thiol ratios were lower compared with GA, especially at 6 h and 18 h postoperatively (*p* = 0.007 and *p* = 0.019, respectively). No significant differences were observed in C-reactive protein (CRP) levels between groups (*p* > 0.05).

The intra-group analysis revealed that in the GA group, thiol levels decreased, while in the IB group, native and total thiols increased postoperatively (*p* = 0.032 and *p* = 0.012, respectively), indicating a better preserved antioxidant capacity after regional anesthesia [35].

The results of another study on brachial plexus blockade, this time via the axillary approach during unilateral hand or forearm surgery, confirm a positive correlation between this blockade and oxidative status. Compared to TIVA (propofol/remifentanil) and inhalational anesthesia (sevoflurane), axillary block group showed the most favorable oxidative profile, with significantly lower ischemia-modified albumin (IMA) and total oxidant status (TOS) values at all post-ischemic time points (*p* < 0.001 and *p* < 0.01, respectively), although TAS levels were higher with inhalational anesthesia. The authors suggested that this antioxidative effect observed under axillary brachial plexus block was likely due to sympathetic blockade and improved microcirculatory perfusion, which enhance local oxygen delivery and facilitate the removal of reactive oxygen species [36].

### 4.3. Pediatric Studies

Regarding pediatric patients, the number of available studies is highly limited. Two articles from a single study conducted in Serbia were identified. In a clinical study involving 45 children aged 8–17 years undergoing extremity surgery with tourniquet application, Budic et al. [37] investigated the impact of three anesthetic techniques—sevoflurane inhalation, TIVA with propofol, and regional anesthesia—on oxidative stress biomarkers. Blood samples were taken before and after tourniquet release, and oxidative stress was assessed using plasma and erythrocyte levels of malondialdehyde (MDA), xanthine oxidase (XO), and antioxidant enzymes. The sevoflurane group showed the highest increase in MDA and XO levels, particularly after reperfusion, indicating enhanced lipid peroxidation and oxidative stress. In contrast, TIVA and regional anesthesia significantly limited MDA accumulation and better preserved antioxidant status. These findings suggest that volatile anesthesia with sevoflurane exacerbates ischemia–reperfusion-induced oxidative stress, while propofol-based TIVA and regional techniques provide more effective antioxidant protection in pediatric patients [37,38].

The most recent study published in 2025 focused on the role of regional anesthesia—specifically peripheral nerve blocks—in the context of ischemia–reperfusion injury caused by tourniquet application in children aged 6–12 undergoing knee arthroscopy. The results of the research demonstrated a potential effect of adding regional block to general anesthesia in mitigating oxidative stress–induced tissue injury. In this study, the group of 60 children who received general anesthesia combined with a femoral nerve block and sciatic nerve block showed significantly lower MDA levels and higher SOD activity at 60 min after tourniquet inflation and 30 min after its release, compared to the control group that underwent arthroscopy only under general anesthesia [39].

### 4.4. Gynecology and Obstetrics

Gynecology and obstetrics, although considered a single specialization, is broad and includes a wide range of surgical procedures. When it comes to obstetrics, cesarean section is the most common surgical intervention. Numerous cohort studies and meta-analyses suggest the superiority of regional anesthesia over general anesthesia and recommend, whenever possible, the use of neuraxial techniques in both elective and emergency cesarean sections, as spinal anesthesia provides greater maternal safety, more stable hemodynamics, reduced risk of airway complications, and improved neonatal outcomes [40,41]. The aspect of oxidative stress connected to the anesthesia used during the delivery has not been well examined. While compiling the bibliography for this review, only 3 original articles were found.

One article described a study among 47 parturients, focusing on fetal antioxidant and oxidant status by investigating the levels of OSI, TAS, and TOS in umbilical arterial blood samples. The patients were divided into 3 groups depending on the chosen anesthesia technique (spinal, epidural or general). The levels of TAS and TOS were not statistically different (*p* = 0.448 and *p* = 0.050) among the three groups, but OSI values in the group with general anesthesia were lower when compared with those in the epidural anesthesia group. In this study, maternal oxidative status was not investigated, as the analysis was restricted to umbilical cord arterial blood, thereby reflecting only fetal oxidative balance [42].

Karam and Mohammad (2023) [43] evaluated maternal oxidative stress in 102 women undergoing elective cesarean section under general anesthesia with propofol or spinal anesthesia with bupivacaine. Maternal blood was sampled before and immediately after surgery. Both groups showed a significant reduction in plasma malondialdehyde (MDA) (−13% in GA and −24% in SA, *p* < 0.05), whereas total antioxidant status (TAS) remained unchanged. The authors concluded that both anesthetic techniques reduced oxidative stress, but spinal anesthesia produced a slightly more favorable antioxidant. In this study, only maternal oxidative status was evaluated, as all biochemical measurements were performed on blood samples obtained from the mothers, with no assessment of neonatal oxidative parameters [43].

Oxidative stress as an effect of spinal and general anesthesia techniques used during cesarean section was also examined with another biomarker—maternal–neonatal thiol–disulfide homeostasis. In this study, the oxidative status of both mothers and their newborns was evaluated. Blood samples were collected preoperatively and postoperatively from 80 parturient and from the umbilical venous cords after delivery. Neonates in the general anesthesia group had lower native and total thiol levels compared to spinal anesthesia in group when compared with Group S (*p* = 0.01 and *p* = 0.003). Also maternal disulfide levels after operation were significantly higher in the general anesthesia group and native thiol and total thiol levels were significantly reduced in general anesthesia group compared to spinal anesthesia group [44].

Apart from obstetrics, spinal anesthesia in gynecology laparoscopy is typically avoided due to adverse effects caused by pneumoperitoneum, which is poorly tolerated by awake patients. Trendelenburg position can reduce pulmonary compliance, leading to hypercapnia, which makes spinal anesthesia more challenging [45]. Moreover, the comparison of general and spinal anesthesia regarding oxidative stress showed no significant difference in terms of total antioxidant capacity (TAC), total oxidant level (TOL), and oxidative stress index (OSI) (*p* = 0.862, *p* = 0.940, and *p* = 0.728, respectively). The described study was unique in the sense that the effects of SA and GA on oxidative–antioxidative status in gynecologic laparoscopic surgery were evaluated. Because of the limited amount of data, more studies are needed to determine the impact of regional anesthesia on oxidative stress in gynecology procedures [46].

In the table below (Table 2), the key points of the research studies cited in this review are presented.

### 4.5. Animal Studies

In our review, we decided to include animal studies as well, since they provide essential mechanistic evidence regarding the effects of anesthesia on oxidative stress. These experimental models allow for controlled investigation of biochemical and cellular processes that cannot be directly assessed in human subjects. One of the latest studies using an animal model highlights the positive impact of spinal anesthesia on the response to oxidative stress. In this investigation regarding reconstructive surgery (muscle-skin flap surgeries using rat models), spinal anesthesia induced lower levels of MDA and TOS as well as higher TAS compared to general anesthesia [47].

Another animal study evaluating the effects of spinal anesthesia with bupivacaine is reported in the recent work by Liu et al. (2025) [48], which investigated the impact of spinal bupivacaine on myocardial injury and underlying cellular mechanisms in 72 rats undergoing thoracic surgery. In this study, male Sprague Dawley rats were divided into four groups: Control, Sham (spinal saline), Surgery (saline + thoracic incision), and Bupivacaine (spinal bupivacaine + thoracic incision). The study showed that spinal anesthesia with bupivacaine significantly reduced serum markers of myocardial injury (cTnI, CK-MB, LD), decreased oxidative stress (lower MDA, higher SOD and GPX), and inhibited cardiomyocyte apoptosis compared to the Surgery group. Histological and ultrastructural analyses confirmed reduced myocardial damage and preserved mitochondrial integrity in the Bupivacaine group. These results support the cardioprotective role of spinal bupivacaine in the context of surgical stress through antioxidant and anti-apoptotic mechanisms [48].

## 5. Discussion

Modern anesthesiology requires specialists to choose the appropriate type of anesthesia tailored to each individual patient, their comorbidities, and the type of surgery. The anesthesiologist should consider each case individually, weighing the advantages and disadvantages of each technique, as well as the potential benefits and anticipated challenges associated with the chosen method—not only in the perioperative period but also in terms of its long-term impact on the patient’s future. Despite the rapid advancement of medicine, relatively little is still known about the long-term effects of anesthesia, particularly with respect to regional anesthesia. This review sought to identify studies on the effect of regional anesthesia on oxidative stress.

Research on oxidative stress clearly demonstrates the impact of an imbalance between reactive oxygen species and the body’s biological capacity to neutralize them on the epidemiology of certain chronic diseases. However, it remains unclear whether oxidative stress induced by anesthesia is strong enough to influence the development of such diseases. Nevertheless, several studies have highlighted the impact of oxidative stress on perioperative outcomes, including the patient’s physiological stability, recovery process, inflammatory response, and wound healing [49,50,51,52].

This review analyzed studies comparing regional and general anesthesia in terms of oxidative stress biomarkers. Overall, the evidence remains heterogeneous. While several studies suggest that regional anesthesia (RA), particularly spinal and peripheral nerve blocks, may attenuate oxidative stress by reducing sympathetic activation and improving microcirculatory perfusion, others have demonstrated equivalent or even lower oxidative stress levels under general anesthesia (GA), particularly with propofol-based total intravenous anesthesia (TIVA). These discrepancies highlight the complexity of redox regulation in the perioperative period and the multifactorial nature of oxidative stress responses.

From a mechanistic standpoint, regional anesthesia may confer antioxidant advantages through decreased catecholamine release, improved oxygen delivery, and reduced ischemia–reperfusion injury. In obstetric and cesarean section studies, RA was associated with more favorable maternal and neonatal redox profiles compared with GA, possibly due to lower systemic stress and better hemodynamic stability. Similarly, in orthopedic surgery, regional blocks or neuraxial techniques were often linked to reduced lipid peroxidation and preservation of antioxidant defenses. However, this advantage was not universal: studies involving tourniquet use or high FiO_2_ exposure frequently demonstrated higher oxidative markers under RA. Conversely, several investigations showed that GA with propofol or sevoflurane decreased oxidative biomarkers such as MDA and IMA, reflecting the intrinsic antioxidant properties of these agents. This suggests that the type of anesthetic drug may play a more decisive role than the choice of anesthetic technique itself.

In addition to differences in anesthetic pharmacology, many intraoperative factors can modulate oxidative balance. Variables such as the use and duration of tourniquet application, the extent of tissue trauma and bleeding, intraoperative FiO_2_ concentration, and hemodynamic fluctuations can all influence redox outcomes. Individual anesthesiologist preferences, variability in analgesic protocols, and perioperative temperature or fluid management may further confound results. Consequently, attributing observed oxidative changes solely to the anesthetic technique is inherently challenging.

The existing evidence base is further limited by methodological heterogeneity. Most studies included small patient populations (often fewer than 100 participants), short observation periods, and non-randomized designs. The diversity of biomarkers used—ranging from malondialdehyde (MDA) and F_2_-isoprostanes to thiol/disulfide ratios and gene expression analyses—also hinders direct comparison. Moreover, various surgical disciplines differ significantly in baseline oxidative burden; for instance, orthopedic procedures involving ischemia–reperfusion injury or obstetric surgeries under physiological stress of pregnancy cannot be directly compared.

There is also a clear lack of studies evaluating the isolated impact of individual anesthetic agents on oxidative stress. Anesthesia is typically multimodal, combining multiple drugs such as volatile anesthetics, intravenous agents, opioids, and adjuvants, making it difficult to separate the contribution of each compound. Future studies should therefore control for pharmacologic confounders and focus on standardized redox biomarker panels measured at multiple perioperative time points.

Taken together, the current evidence cautiously supports the notion that regional anesthesia may offer a more favorable oxidative stress profile in selected settings—particularly in obstetric and lower-limb orthopedic procedures—although the data remain inconsistent. The variability of findings likely reflects differences in anesthetic drug composition, surgical stress, and methodological design. Future large-scale, randomized, and standardized trials are needed to confirm these observations, delineate the molecular mechanisms underlying anesthetic-induced redox modulation, and determine whether these biochemical effects translate into clinically meaningful outcomes. A better understanding of these processes may pave the way toward safer, more personalized, and physiologically tailored anesthesia in the future [53].

## Figures and Tables

**Table 1:** Biomarkers of oxidative stress most frequently evaluated in studies.

Type of Oxidative Stress Biomarker	Name of Oxidative Stress Biomarker	Biological Function	Expected Change Under OS
Lipid Peroxidation Products	MDA	Oxidative damage to cell membranes	↑
	Isofurane (IsoF)	Markers of lipid peroxidation and oxidative stress under normal oxygen levels	↑
	F2-Isoprostanes	Lipid peroxidation markers formed preferentially under high oxygen tension	↑
Protein Oxidation Products	Protein Carbonyls	Markers of oxidative protein damage and irreversible protein oxidation	↑
	Total Thiol (TT)	Reflects overall antioxidant capacity of plasma thiol groups	↓
	Native Thiol (NT)	Represents reduced thiol groups with direct antioxidant activity	↓
	Disulfide Levels (DS)	Indicate oxidized form of thiols; increase reflects oxidative stress	↑
	Thiol-Disulfide Ratio (NT/DS):	Dynamic indicator of redox balance between antioxidants and oxidants	↓
Indicators of Antioxidant Status	Catalase (CAT)	Antioxidant enzyme whose activity reflects cellular response to hydrogen peroxide–mediated oxidative stress	↑
	Glutathione Peroxidase (GPX)	Antioxidant enzyme that reduces peroxides; its activity indicates cellular defense against oxidative stress	↓
Global oxidative stress indicators	TAC (Total Antioxidant Capacity)	Reflects the overall ability of plasma to neutralize free radicals	↓
	TAS (Total Antioxidant Status)	Cumulative measure of all antioxidants present in plasma	↓
	TOS (Total Oxidant Status)	Indicates the total concentration of oxidants; elevated levels reflect increased oxidative stress	↑
	OSI (Oxidative Stress Index)	Ratio of TOS to TAS; represents the balance between oxidants and antioxidants	↑

**Table 2:** Changes in biomarkers of oxidative stress.

Surgery	Type of Anesthesia	Number of Participants	Oxidative Stress Biomarker	Result
Knee replacement surgery [30]	GA vs. SA	39	F2-IsoPs and IsoFs	SA: lower plasma levels of IsoFs GA: lower plasma levels of F2-IsoPs
Arthroscopic knee surgery [28]	SA vs. TIVA vs. GA (Sevoflurane)	60	MDA	TIVA: the lowest MDA levels
Lower Limb Amputation Surgery [31]	SA vs. GA		MDA, TAC	SA: MDA decrease more significant GA: TAC decrease more significant
		40		
Hip arthroplasty [34]	GA vs. RA (lumbar plexus block) vs. Integrated anesthesia (lumbar plexus block + spinal anesthesia + general anesthesia)	99	gene expression profiles involved in oxidative stress (SOD, GPX)	RA: reduced activation of oxidative stress-related genes compared to general anesthesia
ORIF [32]	GA vs. SA	40	MDA, glutathione, catalase	GA: increase in postoperative MDA levels reduction in catalase activity
Shoulder arthroscopy [35]	Interscalene block vs. GA	42	Native thiol, total thiol, disulphide	IB: higher native thiol and total thiol levels, lower levels of disulphide
Knee Replacement Surgery [29]	SA vs. GA	56	Native thiol, total thiol, disulphide	GA: higher Native Thiol and Total Thiol Levels. SA: higher Disulfide Levels. Thiol/Disulfide Ratios: Favorable in the GA
Unilateral hand or forearm surgery [36]	TIVA vs. inhalation anesthesia vs. brachial plexus block	99	TAS, TOS, IMA	Axillary Block: Showed the lowest oxidative stress markers (IMA and TOS) Inhalation Anesthesia: higher TAS levels
Lower and upper limb operations [38]	GA vs. TIVA vs. RA	45	MDA, xanthine oxidase, protein carbonyl groups	Protein Carbonyl Significantly higher in GA group 20 min after reperfusion. MDA: highest levels were observed in the GA group
Lower and upper limb operations [37]	GA vs. TIVA vs. RA	45	MDA, catalase	CAT activity increased in the GA and RA groups but significantly decreased in TIVA group during reperfusion
Knee arthroscopic surgery [39]	GA vs. GA + RA (femoral nerve block combined with sciatic nerve block)	64	MDA, SOD	RA: lower MDA levels and higher SOD activity after tourniquet release
Total knee arthroplasty [33]	GA vs. GA + FNB	297	MDA, GPX	RA: ↓ MDA, ↓ GPX
Robot assisted laparoscopic prostatectomy [26]	GA + epidural vs. GA	45	MDA	Increase in MDA levels in group GA
Midline laparotomy [22,23,24]	RSB + GA vs. GA	56	Glutathione Peroxidase	No significant increase in oxidative stress biomarkers caused by RSB
Inguinal hernia surgery [25]	GA vs. SA	52	Dynamic Thiol/Disulfide Homeostasis	No significant differences in oxidative stress levels (thiol/disulfide balance) between this two methods
Laparoscopy [46]	SA vs. GA	60	TAC, TOL, OSI	No significant difference was observed between SA and GA
Cesarean sections [42]	SA vs. GA vs. epidural	47	TAS, TOS, OSI	**TAS and TOS**: No statistically significant difference between the groups
Cesarean sections [44]	GA vs. SA	80	Native thiol, total thiol, disulfide	Neonates in the GA showed **lower native and total thiol levels**, increased postoperative maternal **disulfide levels** in GA group. Native thiol and total thiol levels reduced in mothers under GA
Cesarean sections [43]	GA vs. SA	102	MDA, TAS	**MD**A decreased significantly in both groups post-surgery; more pronounced reduction in the SA No significant change in **TAS** in both groups

## Data Availability

No new data were created or analyzed in this study. Data sharing is not applicable to this article.

## Abbreviations

The following abbreviations are used in this manuscript:

8-OHdG	8-Hydroxy-2′-deoxyguanosine
ABTS	2,2′-Azino-bis(3-ethylbenzothiazoline-6-sulfonic acid)
ASA	American Society of Anesthesiologists
CAT	Catalase
COPD	Chronic Obstructive Pulmonary Disease
CRP	C-reactive Protein
cTnI	Cardiac Troponin I
DNPH	2,4-Dinitrophenylhydrazine
DS	Disulfide
EGR1	Early Growth Response 1
ERCC3	Excision Repair Cross-Complementation Group 3
FiO_2_	Fraction of Inspired Oxygen
F_2_-IsoP/F_2_-IsoPs	F_2_-Isoprostanes
FNB	Femoral Nerve Block
GA	General Anesthesia
GC-MS	Gas Chromatography–Mass Spectrometry
GPX/GPX1	Glutathione Peroxidase/Glutathione Peroxidase 1
GR	Glutathione Reductase
GSH	Reduced Glutathione
GSSG	Glutathione Disulfide
H_2_O_2_	Hydrogen Peroxide
HSF1	Heat Shock Factor 1
hs-CRP	High-sensitivity C-reactive Protein
IB	Interscalene Block
IMA	Ischemia-Modified Albumin
IsoF/IsoFs	Isofurans
LD	Lactate Dehydrogenase
MDA	Malondialdehyde
NADPH	Nicotinamide Adenine Dinucleotide Phosphate (reduced form)
NO•	Nitric Oxide
NO_2_^−^	Nitrite
NT	Native Thiol
ONOO^−^	Peroxynitrite
ORIF	Open Reduction and Internal Fixation
OSI	Oxidative Stress Index
O_2_•^−^	Superoxide Anion
PONV	Postoperative Nausea and Vomiting
QoR-15C	Quality of Recovery–15 Questionnaire (Chinese version)
RA	Regional Anesthesia
RNB	Regional Nerve Block
RNS	Reactive Nitrogen Species
ROS	Reactive Oxygen Species
RSB	Rectus Sheath Block
SA	Spinal Anesthesia
SOD/SOD1	Superoxide Dismutase/Superoxide Dismutase 1
SS	Disulfide
TAC	Total Antioxidant Capacity
TAS	Total Antioxidant Status
TIVA	Total Intravenous Anesthesia
TNB	5-thio-2-nitrobenzoic acid
TOL	Total Oxidant Level
TOS	Total Oxidant Status
TT	Total Thiol
VAS	Visual Analogue Scale
XRCC1	X-ray Repair Cross-Complementing Protein 1
XO	Xanthine Oxidase

## References

[1] Jones D.P. (2006). Redefining oxidative stress. Antioxid. Redox Signal..

[2] Phaniendra A., Jestadi D.B., Periyasamy L. (2015). Free radicals: Properties, sources, targets, and their implication in various diseases. Indian J. Clin. Biochem..

[3] Finkel T. (2011). Signal transduction by reactive oxygen species. J. Cell Biol..

[4] García-Sánchez A., Miranda-Díaz A.G., Cardona-Muñoz E.G. (2020). The Role of Oxidative Stress in Physiopathology and Pharmacological Treatment with Pro- and Antioxidant Properties in Chronic Diseases. Oxidative Med. Cell. Longev..

[5] Reddy V.P. (2023). Oxidative Stress in Health and Disease. Biomedicines.

[6] Braz M.G., Braz L.G., Freire C.M.M., Lucio L.M.C., Braz J.R.C., Tang G., Salvadori D.M.F., Yeum K.J. (2015). Isoflurane and Propofol Contribute to Increasing the Antioxidant Status of Patients During Minor Elective Surgery: A Randomized Clinical Study. Medicine.

[7] Kundović S.A., Rašić D., Popović L., Peraica M., Črnjar K. (2020). Oxidative stress under general intravenous and inhalation anaesthesia. Arch. Ind. Hyg. Toxicol..

[8] Abilés J., de la Cruz A.P., Castaño J., Rodríguez-Elvira M., Aguayo E., Moreno-Torres R., Llopis J., Aranda P., Argüelles S., Ayala A. (2006). Oxidative stress is increased in critically ill patients according to antioxidant vitamins intake, independent of severity: A cohort study. Crit. Care.

[9] Senoner T., Velik-Salchner C., Luckner G., Tauber H. (2021). Anesthesia-Induced Oxidative Stress: Are There Differences between Intravenous and Inhaled Anesthetics?. Oxidative Med. Cell. Longev..

[10] Bedirli N., Akyürek N., Kurtipek O., Kavutcu M., Kartal S., Bayraktar A.C. (2011). Thoracic epidural bupivacaine attenuates inflammatory response, intestinal lipid peroxidation, oxidative injury, and mucosal apoptosis induced by mesenteric ischemia/reperfusion. Anesth. Analg..

[11] Derindağ G., Akgül H.M., Kızıltunç A., Özkan H., Kızıltunç Özmen H., Akgül N. (2021). Evaluation of saliva glutathione, glutathione peroxidase, and malondialdehyde levels in head-neck radiotherapy patients. Turk. J. Med Sci..

[12] Jhang J.F., Yu W.R., Huang W.T., Kuo H.C. (2024). Combination of urinary biomarkers and machine-learning models provided a higher predictive accuracy to predict long-term treatment outcomes of patients with interstitial cystitis/bladder pain syndrome. World J. Urol..

[13] Mori T.A., Croft K.D., Puddey I.B., Beilin L.J. (1999). An improved method for the measurement of urinary and plasma F2-isoprostanes using gas chromatography-mass spectrometry. Anal. Biochem..

[14] Levine R.L., Garland D., Oliver C.N., Amici A., Climent I., Lenz A.G., Ahn B.W., Shaltiel S., Stadtman E.R. (1990). Determination of carbonyl content in oxidatively modified proteins. Methods Enzymol..

[15] Dalle-Donne I., Rossi R., Giustarini D., Milzani A., Colombo R. (2003). Protein carbonyl groups as biomarkers of oxidative stress. Clin. Chim. Acta.

[16] Poimenova I.A., Sozarukova M.M., Ratova D.V., Nikitina V.N., Khabibullin V.R., Mikheev I.V., Proskurnina E.V., Proskurnin M.A. (2024). Analytical Methods for Assessing Thiol Antioxidants in Biological Fluids: A Review. Molecules.

[17] Silvestrini A., Meucci E., Ricerca B.M., Mancini A. (2023). Total Antioxidant Capacity: Biochemical Aspects and Clinical Significance. Int. J. Mol. Sci..

[18] Erel O. (2004). A novel automated direct measurement method for total antioxidant capacity using a new generation, more stable ABTS radical cation. Clin. Biochem..

[19] Erel O. (2005). A new automated colorimetric method for measuring total oxidant status. Clin. Biochem..

[20] De Cassai A., Sella N., Geraldini F., Tulgar S., Ahiskalioglu A., Dost B., Manfrin S., Karapinar Y.E., Paganini G., Beldagli M. (2023). Single-shot regional anesthesia for laparoscopic cholecystectomies: A systematic review and network meta-analysis. Korean J. Anesthesiol..

[21] Baloyiannis I., Perivoliotis K., Sarakatsianou C., Tzovaras G. (2018). Laparoscopic total extraperitoneal hernia repair under regional anesthesia: A systematic review of the literature. Surg. Endosc..

[22] Purdy M., Kokki M., Anttila M., Aspinen S., Juvonen P., Selander T., Kokki H., Pulkki K., Eskelinen M. (2016). Does Post-Surgery Placement of Rectus Sheath Block Analgesia Alter the Oxidative Stress Biomarker 8-OHdG Concentrations: A Randomised Trial of Patients with Cancer and Benign Disease. Cancer Genom. Proteom..

[23] Purdy M., Kärkkäinen J., Kokki M., Anttila M., Aspinen S., Juvonen P., Kokki H., Pulkki K., Rantanen T., Eskelinen M. (2017). Does Rectus Sheath Block Analgesia Alter Levels of the Oxidative Stress Biomarker Glutathione Peroxidase: A Randomised Trial of Patients with Cancer and Benign Disease. Anticancer Res..

[24] Kärkkäinen J., Selander T., Purdy M., Juvonen P., Eskelinen M. (2018). Patients with Increased Levels of the Oxidative Stress Biomarker SOD1 Appear to Have Diminished Postoperative Pain After Midline Laparotomy: A Randomised Trial with Special Reference to Postoperative Pain Score (NRS). Anticancer. Res..

[25] Çelik F., Dağlı R., İlanbey B. (2024). Comparison of the effects of Anesthesia Methods on Dynamic Thiol/Disulfide Homeostasis in Patients with Chronic Obstructive Pulmonary Disease Under Surgical Stress. Ahi Evran Med. J..

[26] Shin S., Bai S.J., Rha K.H., So Y., Oh Y.J. (2013). The effects of combined epidural and general anesthesia on the autonomic nervous system and bioavailability of nitric oxide in patients undergoing laparoscopic pelvic surgery. Surg. Endosc..

[27] Greimel F., Maderbacher G., Baier C., Keshmiri A., Schwarz T., Zeman F., Meissner W., Grifka J., Benditz A. (2018). Multicenter cohort-study of 15326 cases analyzing patient satisfaction and perioperative pain management: General, regional and combination anesthesia in knee arthroplasty. Sci. Rep..

[28] Koşucu M., Coşkun I., Eroglu A., Kutanis D., Menteşe A., Karahan S.C., Baki E., Kerimoğlu S., Topbas M. (2014). The effects of spinal, inhalation, and total intravenous anesthetic techniques on ischemia-reperfusion injury in arthroscopic knee surgery. BioMed Res. Int..

[29] Simsek E.M., Aksoy S.M., Manti N., Erel O., Neselioglu S., Firat A. (2023). The Effect of Spinal and General Anesthesia on Thiol-Disulfide Balance During Ischemia/Reperfusion of the Leg in Patients Undergoing Knee Replacement Surgery. J. Anesth. /Anestezi Derg..

[30] Mas E., Barden A.E., Corcoran T.B., Phillips M., Roberts L.J., Mori T.A. (2011). Effects of spinal or general anesthesia on F_2_-isoprostanes and isofurans during ischemia/reperfusion of the leg in patients undergoing knee replacement surgery. Free. Radic. Biol. Med..

[31] Peivandi Yazdi A., Bameshki A., Salehi M., Kazemzadeh G., Sharifian Razavi M., Rahmani S., Hashemy S.I. (2018). The Effect of Spinal and General Anesthesia on Serum Lipid Peroxides and Total Antioxidant Capacity in Diabetic Patients with Lower Limb Amputation Surgery. Arch. Bone Jt. Surg..

[32] Aremu P.A., Ajayi A.M., Ben-Azu B., Orewole O.T., Umukoro S. (2020). Spinal and general anesthesia produces differential effects on oxidative stress and inflammatory cytokines in orthopedic patients. Drug Metab. Pers. Ther..

[33] Zhao L., Qiu D. (2024). Ultrasound-guided femoral nerve block reduced the incidence of postoperative delirium after total knee arthroplasty: A double-blind, randomized study. Medicine.

[34] Alleva R., Tognù A., Tomasetti M., Benassi M.S., Pazzaglia L., van Oven H., Viganò E., De Simone N., Pacini I., Giannone S. (2019). Effect of different anaesthetic techniques on gene expression profiles in patients who underwent hip arthroplasty. PLoS ONE.

[35] Oksuz M., Abitagaoglu S., Kaciroglu A., Koksal C., Ozturk B.Y., Erel O., Senat A., Erdogan Ari D. (2021). Effects of general anaesthesia and ultrasonography-guided interscalene block on pain and oxidative stress in shoulder arthroscopy: A randomised trial. Int. J. Clin. Pract..

[36] Kutanis D., Erturk E., Akdogan A., Besir A., Altinbas A., Orem A., Kara H., Yıldız M., Mentese A. (2024). Comparison of the effects of axillary brachial plexus block, inhalation anesthesia, and total intravenous anesthesia on tourniquet-induced ischemia-reperfusion injury in upper extremity surgery. Turk. J. Trauma Emerg. Surg..

[37] Budić I., Pavlović D., Cvetković T., Djordjević N., Simić D., Milojević I., Stojanović M. (2010). The effects of different anesthesia techniques on free radical production after tourniquet-induced ischemia-reperfusion injury at children’s age. Vojnosanit. Pregl..

[38] Budic I., Pavlovic D., Kocic G., Cvetkovic T., Simic D., Basic J., Zivanovic D. (2011). Biomarkers of oxidative stress and endothelial dysfunction after tourniquet release in children. Physiol. Res..

[39] Xia Q., Chen R., Lin C., Zhang B., Zhou L., Xiong J., Lv Z., Mao Y., Ren R. (2025). Effect of regional nerve block on tourniquet-related injury in pediatric patients undergoing lower limb surgery: A randomized controlled study. Ann. Med..

[40] Shi X., Xu C., Wen Y., Jiang M., Yu H., Wang X., Yuan H., Feng S. (2024). Perinatal outcome of emergency cesarean section under neuraxial anesthesia versus general anesthesia: A seven-year retrospective analysis. BMC Anesthesiol..

[41] Sung T.Y., Jee Y.S., You H.J., Cho C.K. (2021). Comparison of the effect of general and spinal anesthesia for elective cesarean section on maternal and fetal outcomes: A retrospective cohort study. Anesth. Pain Med..

[42] Karabayırlı S., Keskin E.A., Kaya A., Koca C., Erel O., Demircioglu R.I., Muslu B. (2015). Assessment of fetal antioxidant and oxidant status during different anesthesia techniques for elective cesarean sections. J. Res. Med Sci..

[43] Karam R.S., Mohammad F.K. (2023). Changes in blood oxidative stress biomarker and cholinesterase activity after general vs spinal anesthesia for elective cesarean sections. Anaesth. Pain Intensiv. Care.

[44] Akin F., Kozanhan B., Deniz C.D., Sahin O., Goktepe H., Neselioglu S., Erel O. (2019). Effects of the anesthesia technique used during cesarean section on maternal-neonatal thiol disulfide homeostasis. Minerva Anestesiol..

[45] Pusapati R.N., Sivashanmugam T., Ravishankar M. (2010). Respiratory changes during spinal anaesthesia for gynaecological laparoscopic surgery. J. Anaesthesiol. Clin. Pharmacol..

[46] Kaya Uğur B., Pirbudak L., Öztürk E., Balat Ö., Uğur M.G. (2020). Spinal versus general anesthesia in gynecologic laparoscopy: A prospective, randomized study. J. Turk. Soc. Obstet. Gynecol..

[47] Akkoç M.F., Kapı E., Bozkurt M., Işık F.B., Çelik Y. (2022). Comparison of the Effects of Inhalation and Spinal Anesthesia on Microcirculation in Transverse Rectus Abdominis and Gluteus Maximus Muscle-Skin Flap Applications in Experimental Rat Models. Gevher Nesibe J. Med. Health Sci..

[48] Liu T.J., Shih V., Yeh Y.C., Lee W.L., Wang L.C., Lai H.C. (2025). Impact of spinal anesthesia on myocardial damage, ultrastructure and cellular mechanisms in rats undergoing thoracic incision surgery. Toxicol. Appl. Pharmacol..

[49] Bruehl S., Milne G., Schildcrout J., Shi Y., Anderson S., Shinar A., Polkowski G., Mishra P., Billings F.T. (2023). Perioperative oxidative stress predicts subsequent pain-related outcomes in the 6 months after total knee arthroplasty. Pain.

[50] Araki O., Matsumura Y., Inoue T., Karube Y., Maeda S., Kobayashi S., Chida M. (2018). Association of Perioperative Redox Balance on Long-Term Outcome in Patients Undergoing Lung Resection. Ann. Thorac. Cardiovasc. Surg..

[51] Geng J., Qian J., Si W., Cheng H., Ji F., Shen Z. (2017). The clinical benefits of perioperative antioxidant vitamin therapy in patients undergoing cardiac surgery: A meta-analysis. Interact. Cardiovasc. Thorac. Surg..

[52] Zheng H., Zheng H., Wei L., Xue Z., Xu B., Hu M., Yu J., Xie R., Zhang L., Zheng Z. (2024). Risk stratification models incorporating oxidative stress factors to predict survival and recurrence in patients with gastric cancer after radical gastrectomy: A real-world multicenter study. Eur. J. Surg. Oncol..

[53] Stevens J.L., Feelisch M., Martin D.S. (2019). Perioperative Oxidative Stress: The Unseen Enemy. Anesth. Analg..

